# Comprehensive development and validation of gene signature for predicting survival in patients with glioblastoma

**DOI:** 10.3389/fgene.2022.900911

**Published:** 2022-08-10

**Authors:** Yi Jin, Zhanwang Wang, Kaimin Xiang, Yuxing Zhu, Yaxin Cheng, Ke Cao, Jiaode Jiang

**Affiliations:** ^1^ Department of Oncology, Third Xiangya Hospital of Central South University, Changsha, China; ^2^ Department of Radiation Oncology, Hunan Cancer Hospital, The Affiliated Cancer Hospital of Xiangya School of Medicine, Central South University, Changsha, China; ^3^ Key Laboratory of Translational Radiation Oncology, Department of Radiation Oncology, Hunan Cancer Hospital and The Affiliated Cancer Hospital of Xiangya School of Medicine, Central South University, Changsha, China; ^4^ Department of Gastroenterological Surgery, The Third Xiangya Hospital, Central South University, Changsha, China; ^5^ Department of Neurosurgery, The Third Xiangya Hospital, Central South University, Changsha, China

**Keywords:** glioblastoma, nomogram, novel classification, WGCNA, signature

## Abstract

Glioblastoma (GBM) is the most common brain tumor, with rapid proliferation and fatal invasiveness. Large-scale genetic and epigenetic profiling studies have identified targets among molecular subgroups, yet agents developed against these targets have failed in late clinical development. We obtained the genomic and clinical data of GBM patients from the Chinese Glioma Genome Atlas (CGGA) and performed the least absolute shrinkage and selection operator (LASSO) Cox analysis to establish a risk model incorporating 17 genes in the CGGA693 RNA-seq cohort. This risk model was successfully validated using the CGGA325 validation set. Based on Cox regression analysis, this risk model may be an independent indicator of clinical efficacy. We also developed a survival nomogram prediction model that combines the clinical features of OS. To determine the novel classification based on the risk model, we classified the patients into two clusters using ConsensusClusterPlus, and evaluated the tumor immune environment with ESTIMATE and CIBERSORT. We also constructed clinical traits-related and co-expression modules through WGCNA analysis. We identified eight genes (*ANKRD20A4, CLOCK, CNTRL, ICA1, LARP4B, RASA2, RPS6*, and *SET*) in the blue module and three genes (*MSH2*, *ZBTB34*, and *DDX31*) in the turquoise module. Based on the public website TCGA, two biomarkers were significantly associated with poorer OS. Finally, through GSCALite, we re-evaluated the prognostic value of the essential biomarkers and verified *MSH2* as a hub biomarker.

## Introduction

Gliomas are the most common primary tumors of the central nervous system, with devastating progression (Ohgaki and Kleihues, 2005). Based on its histological and molecular characteristics, glioblastoma (GBM) is often classified as WHO grade IV (Louis et al., 2021). The absence or presence of mutations in *IDH1* [which encodes isocitrate dehydrogenase (*NADP*)] or *IDH2* [which encodes isocitrate dehydrogenase (*NADP*), mitochondrial], and a 1p and 19q (1p/19q) chromosomal co-deletion may be important biomarkers for GBM diagnosis. Glioblastoma is the most malignant glioma type, with rapid proliferation and fatal invasion, accounting for 48.6% of primary malignant brain tumors, having an annual incidence of 3.23 per 100,000 in the United States (Le Rhun et al., 2019; Ostrom et al., 2021). At present, surgical resection combined with radiotherapy and chemotherapy remains the mainstay of glioma treatment. Some novel treatments, including PD-1 inhibitors, may also improve OS (Lim et al., 2018). Although a great deal of research has focused on improving treatment strategies, glioma patients continue to experience poor disease prognosis, and the median survival time is approximately 14 months (Thorsson et al., 2018). As the mechanisms underlying poor prognosis in GBM patients remain unclear, research aiming to explore these mechanisms and identify potential treatment targets may have great clinical significance.

Up to seven molecular glioblastoma-intrinsic targets involved in oncogenic signaling have been identified. These include tyrosine kinase receptors, cell cycle regulators, and apoptosis-regulating pathway components (Le Rhun et al., 2019). *EGFR,* one of the most prominent oncogenes in GBM, is overexpressed in approximately 60% of tumors, and 40% of tumors exhibit *EGFR* gene amplification (Suina et al., 2018). Numerous studies have failed to demonstrate satisfactory outcomes with *EGFR*-related treatments, including tyrosine kinase inhibitors, vaccines, and rindopepimut (Lassman et al., 2005; Hegi et al., 2011). *TERT* promoter mutations, the most common molecular alteration in GBM, may promote tumor cell immortalization, but have not yet attracted major attention as pharmacological targets for cancer therapy in clinical research (Killela et al., 2013; Takahashi et al., 2019). Notably, several targeted agents for GBM have failed in late clinical development. Therefore, it is essential to identify novel biomarkers and reveal subgroups in which specific therapeutic efficacy could be achieved.

In the present study, we obtained all clinical characteristics and gene expression data from the Chinese Glioma Genome Atlas (CGGA) and defined molecular subtypes of GBM. We developed a prognostic signature comprising potential prognostic genes based on one CGGA cohort and validated this prognostic signature in another CGGA cohort. We also explored the correlation between clinical features and the identified prognostic signature genes utilizing weighted gene co-expression network analysis (WGCNA). Our comprehensive analyses provide new insights into the molecular biomarkers involved in GBM progression.

## Materials and methods

### Data collection

We downloaded the genomic and clinical data of GBM patients from the CGGA database (http://www.cgga.org.cn/). Two CGGA cohorts, including the CGGA325 RNA-seq cohort (http://www.cgga.org.cn, updated on 28 November, 2019) and the CGGA693 RNA-seq cohort (http://www.cgga.org.cn, updated on 28 November, 2019), including gene expression profiles and clinical-pathological data, were selected for this study (Zhao et al., 2021). Samples in all datasets diagnosed with GBM and containing complete clinical information, including survival, age, grade, IDH1 mutational status, and 1p/19q status, were enrolled for a subsequent study. We randomly selected the CGGA693 RNA-seq cohort as the training set and CGGA325 as the validation set. All gene expression levels were normalized between arrays using batch and limma packages (Supplementary Figure S1).

### Selection of potential prognostic biomarkers

Initially, we performed a univariate Cox regression analysis to identify the potential OS in the two CGGA cohorts. Potential prognostic biomarkers in the progression of GBM were considered to have similar prognostic values in both CGGA693 and CGGA325 RNA-seq cohorts. After selecting the overlapping genes, we included all genes with HR > 1 and *p* < 0.05 in our analyses.

### Establishment and evaluation of prognostic risk model in GBM

We developed a prognostic signature by applying LASSO Cox regression (least absolute shrinkage and selection operator) to the CGGA693 RNA-seq cohort (as a training cohort). The risk score was then calculated according to the risk model. The formula for calculating risk score was as follows: risk score = coef1 * gene-1-expression + coef2 * gene-2-expression +. . .coefn * gene-n-expression. Based on the median risk score, CGGA was classified into high and low-risk groups. A Kaplan–Meier (KM) plot was constructed to display the difference in overall survival in the training cohort (CGGA693) and the validation cohort (CGGA325). A heat map was used to visualize the distribution of prognostic gene expression and clinical features. Univariate and multivariate Cox regression analyses were performed to identify the independent prognostic predictors of overall survival. Finally, we established a nomogram to evaluate the clinical value of this prognostic signature.

### Classification of novel subgroups based on the consensus clustering

We identified the optimal clustering number for visualizing the consensus matrix, tracking plot, and cumulative distribution function plot, using the ConsensusClusterPlus R package (Wilkerson and Hayes, 2010) based on prognostic genes from LASSO analysis. Additionally, we adopted three methods to reevaluate the classification: NMDS, tSNE, and PCA (Taguchi and Oono, 2005; Ringnér, 2008; Cieslak et al., 2020). The immune scores, stromal scores, and ESTIMATE scores of GBM were calculated using the “estimate” package (Yoshihara et al., 2013). The abundance of tumor-infiltrating immune cells was evaluated using the CIBERSORT package (http://cibersort.stanford.edu/). Results with *p <* 0.05 obtained from the ESTIMATE algorithm and CIBERSORT analysis were used for further analyses.

### Classification of new subgroups based on WGCNA analysis

WGCNA is a novel method for gene clustering and forming modules with similar expression patterns. To identify clinical traits-related modules, we selected the genes with prognostic value in the Cox analysis, based on the training set, and constructed a correlation network that incorporated important clinical features and genes, using the R package “WGCNA” (Langfelder and Horvath, 2008). Subsequently, we constructed an adjacency matrix to describe the correlation strength between the nodes, and the adjacency matrix was transformed into a topological overlap matrix (TOM). Next, hierarchical clustering was performed to identify modules with at least 30 genes.

### Identification of hub prognostic genes in GBM

Based on the combined results from the two clustering methods, we identified hub prognostic genes for further validation. GEPIA (gepia.cancer-pku.cn/) is a comprehensive and interactive online server that collects cancer microarray data from “The Cancer Genome Atlas” (TCGA) (Tang et al., 2017). We utilized it to validate the hub genes' correlations with overall survival. Furthermore, we explored innate signaling using GSCALite (http://bioinfo.life.hust.edu.cn/web/GSCALite/).

### Cell culture and transfection

The U-118 MG human GBM cell line was purchased from the Procell Life Science and Technology Company (Wuhan, China). U-118 MG cells were maintained in Dulbecco’s modified Eagle’s medium (DMEM; Procell, Wuhan, China) supplemented with 10% fetal bovine serum (FBS; Procell) and 1% penicillin-streptomycin liquid (Procell). U-118 MG cells were cultured in an atmosphere with 5% CO_2,_ at 37°C. Small interfering RNAs (siRNAs) targeting *MSH2* were synthesized by GenePharma (Shanghai, China). Lipofectamine 2000 (Invitrogen, Carlsbad, CA, United States) was used to transfect siRNAs into U-118 MG cells according to the manufacturer’s protocol. The primer sequences of siRNAs targeting MSH2 are listed in Supplementary Table S4.

### Quantitative reverse transcription polymerase chain reaction

TRIzol reagent (TaKaRa Bio, Dalian, China) was used to extract cellular RNAs, according to the manufacturer’s instructions. One microgram of RNA was used to synthesize cDNA using a reverse transcription kit (RR037A; TaKaRa Bio). Quantitative reverse transcription-polymerase chain reaction (qRT-PCR) was performed using a TB Green Premix Ex Taq Kit (RR820A; TaKaRa Bio, Dalian, China). The silencing efficiency of siRNAs was evaluated using the 2^−ΔΔCt^ method. Glyceraldehyde-3-phosphate dehydrogenase (GAPDH) was used as an internal control. The primer sequences for GAPDH and MSH2 are listed in Supplementary Table S4.

### Cell counting kit-8 assay

U-118 MG cell proliferation was assessed using a Cell Counting Kit-8 (CCK-8) kit (Biosharp, Hefei, Anhui, China). U-118 MG cells were transfected with negative control (si-NC) or siRNAs targeting *MSH2*. 10-μL of CCK-8 was added to the cells, and cells were then cultured at 37°C for the indicated days. Optical density at 450 nm (OD_450_) was measured using a microplate spectrophotometer.

### 5-Ethynyl-2′-deoxyuridine (EdU) assay

U-118 MG cells were stained using BeyoClick™ EdU-488 Cell Proliferation Kit (Beyotime, Shanghai, China). In brief, U-118 MG cells (1.5 × 10^5^ cells/well) were seeded in a 6-well plate, transfected with NC or si-MSH2, and cultured in an incubator at 37°C. After 96 h of transfection, U-118 MG cells were incubated with EdU for 3 h, fixed with 1 ml paraformaldehyde (4%) for 15 min, and permeabilized with 0.3% Triton X-100 (Beyotime) for 15 min. Next, the U-118 MG cells were incubated with 500-µL of the click reaction mixture for 30 min in the dark, washed three times, and incubated with Hoechst buffer for 15 min.

### Clone formation assay

U-118 MG cells (3 × 10^5^ cells/well), seeded in 6-well plates, were transfected with si-NC or si-MSH2. Cells were fully trypsinized 24 h after transfection. Cells (*n* = 1,000) were seeded in a 6-well plate and maintained at 37°C for 2–3 weeks. The cells were fixed with paraformaldehyde (4%) for 15 min and stained with crystal violet buffer (Solarbio, Beijing, China) for 30 min.

### Transwell assay

To detect migration and invasion, Transwell chambers (Corning, NY, United States) were prepared uncoated or coated with Matrigel matrix (Corning, NY, United States). U-118 MG cells were collected by trypsinization, 24 h after transfection with different siRNAs. U-118 MG cells (4 × 10^5^ cells/mL) were diluted in a serum-free culture medium and cell suspensions (200 μl, 8 × 10^4^ cells/well) were seeded into the transwell upper chambers. Then, 600 μL DMEM (containing 20% FBS) was added to each lower chamber. The U-118 MG cells were incubated for 36 h. The cells that invaded the chamber membrane were fixed and stained with crystal violet stain buffer (G1073; Solarbio, Beijing, China) for 40 min. Invading cells were counted using an inverted microscope.

### Statistical analyses

Statistical analyses were performed using R software. Cox proportional hazards regression analysis was used to select the independent prognostic genes associated with OS. KM curves were used to compare the clinical outcomes of the subgroups. In all analyses, the statistical *p*-values were bilateral and statistical significance was set at *p* < 0.05.

## Results

### Primary prognostic biomarkers identified by Cox analysis

First, we selected two cohorts: CGGA325 and CGGA693 RNA-seq cohorts with GBM. In the CGGA693 RNA-seq cohort, 235 patients were included in the training set, and 137 patients in CGGA325 were included in the validation set. All clinical data are presented in Supplementary Table S1. After Cox analysis, 718 potential genes were found to be associated with poor overall survival in CGGA693, and 1,682 regulators were associated with poor clinical outcomes in CGGA325 (Supplementary Table S2). Upon selection of the overlapping genes, 31 biomarkers were identified as primary prognostic molecules for constructing the next risk model.

### Prognostic risk model construction and evaluation

**FIGURE 1:**
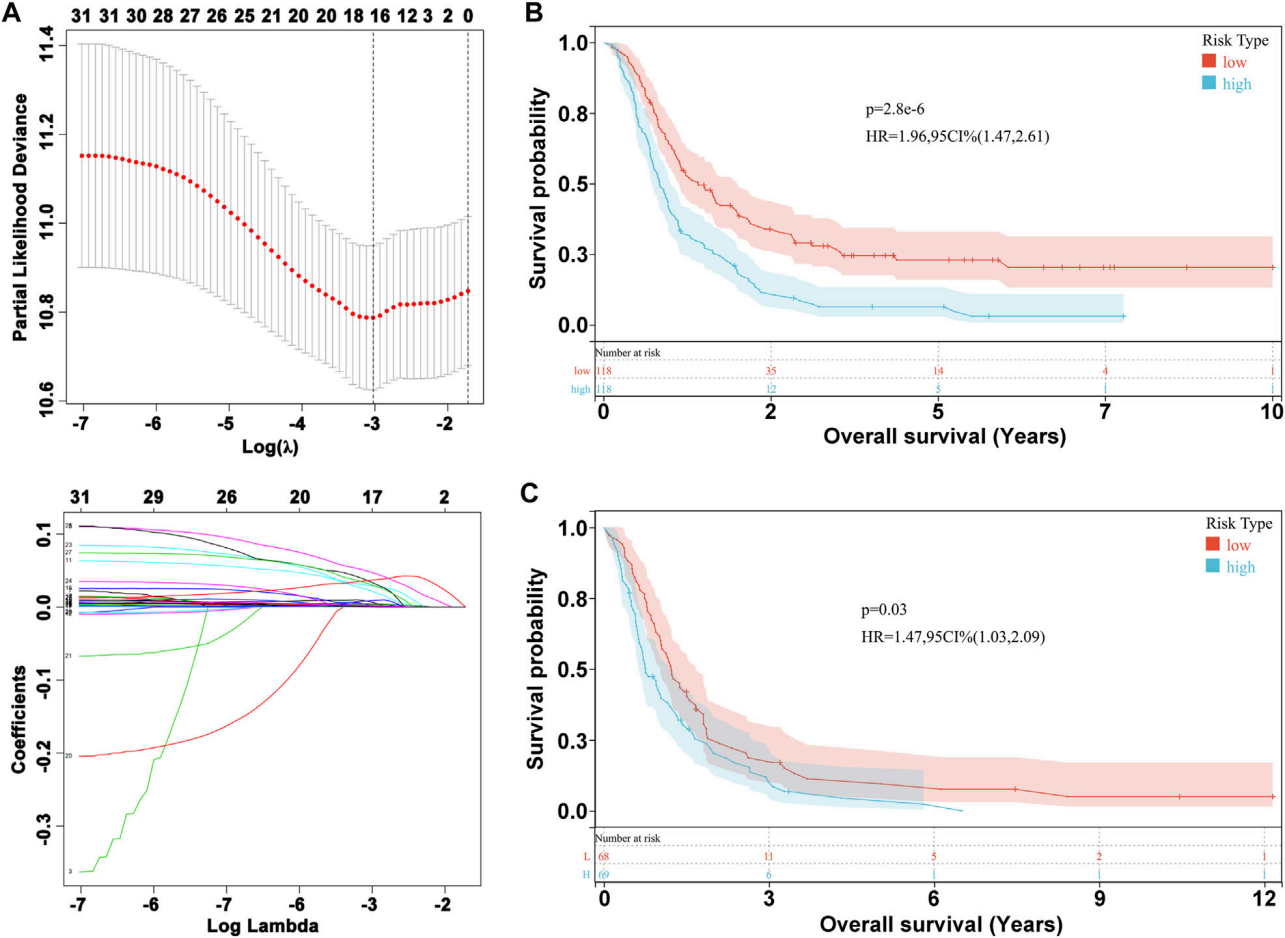
Prognostic risk model construction. **(A)** Evaluation of LASSO Cox analysis. **(B)** KM plot in CGGA693 RNA-seq cohort. **(C)** KM plot in CGGA325 RNA-seq cohort.

**FIGURE 2:**
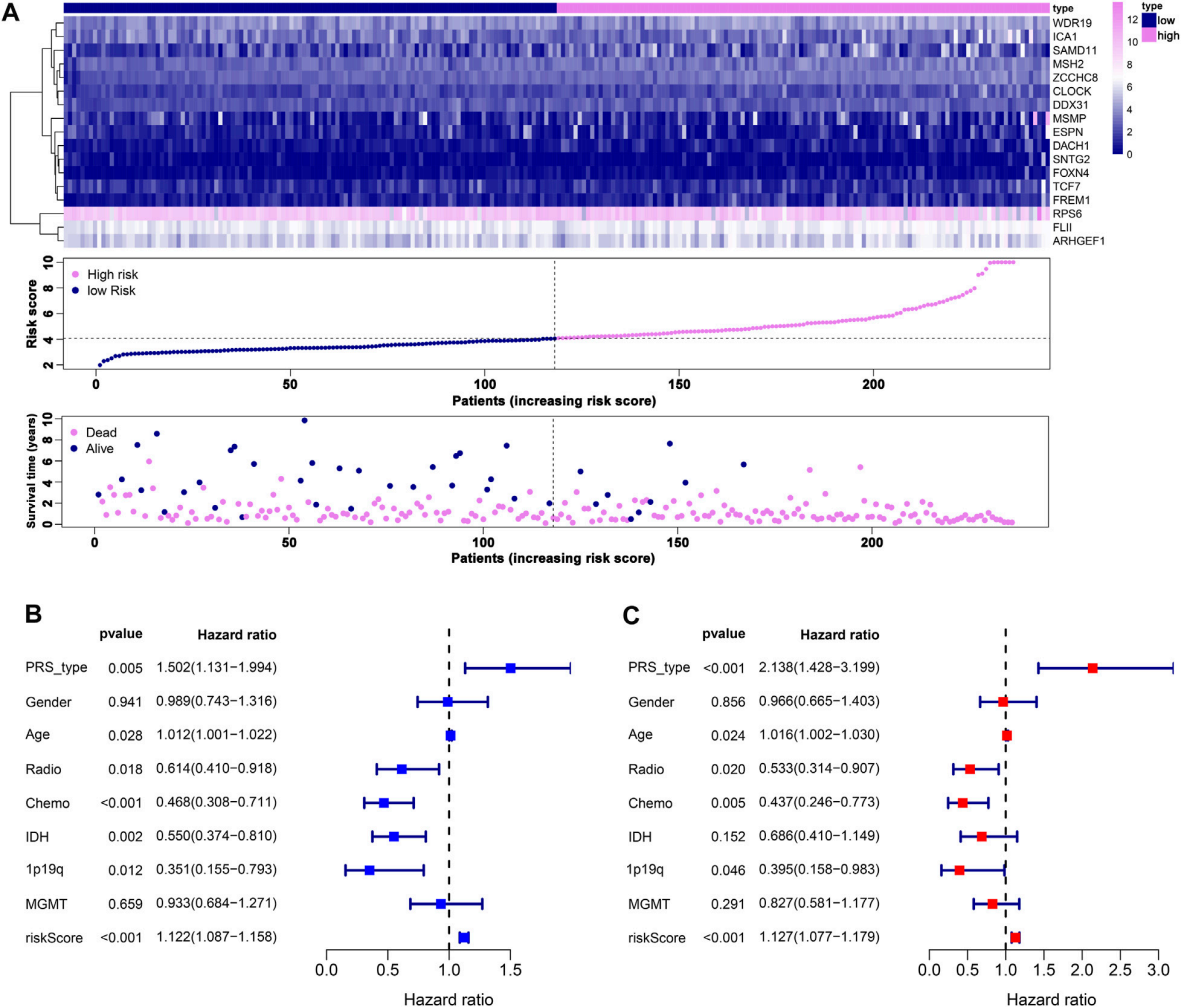
Prognostic risk model estimation. **(A)** Distribution of risk score, survival status, and expression of genes. **(B)** Univariate Cox regression analysis of clinical features and risk score. **(C)** Multivariate Cox regression analysis of clinical features and risk score.

We performed LASSO Cox analysis based on these 31 biomarkers and selected 17 genes to establish a risk model (Figure 1A, Supplementary Table S3), including *MSH2* and *CLOCK*. In the CGGA693 RNA-seq cohort, the KM plot showed that the high-risk score was closely related to poor survival, with *p* < 0.05 (Figure 1B). Similarly, we observed in the validation set that the risk score correlated with poor clinical survival (*p* = 0.04) (Figure 1C). Thus, we constructed a heat map to display the expression profile, distribution of the risk score, patient survival status, and expression pattern of the 17 prognostic genes in the CGGA dataset (Figure 2A). According to univariate and multivariate Cox regression analyses, we found that age and PRS type (primary or recurrent tumor) were correlated with poor survival, and 1p19q status may imply a better clinical outcome. Moreover, treatments such as radiotherapy and chemotherapy may be suitable for improving the prognosis of GBM (Figures 2B,C). The risk score calculated from the prognostic model may be an independent indicator of clinical efficacy. Using this risk score and all clinical characteristics, we developed a survival nomogram prediction model for the OS of patients with GBM using the CGGA dataset (Supplementary Figure S2A). In addition, the calibration curves displayed excellent agreement between observations and predictions in the CGGA dataset (Supplementary Figure S2B).

### Novel classification, construction, and evaluation based on the risk model

**FIGURE 3:**
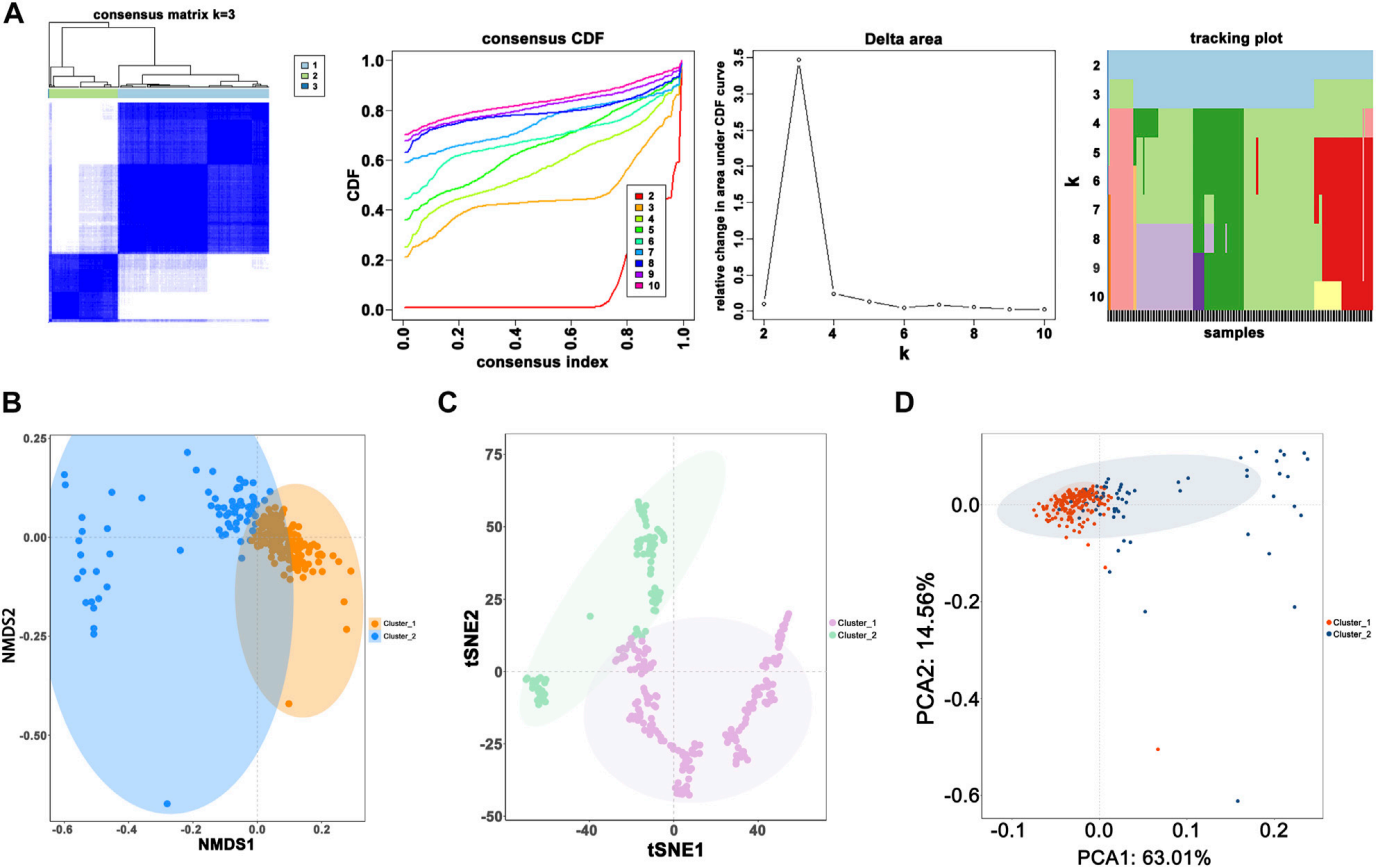
Novel classification construction and evaluation. **(A)** GBM patients were grouped into three clusters according to the consensus clustering matrix (k = 3). **(B)** Re-evaluation of novel classification by NMDS1. **(C)** Re-evaluation of novel classification by tSNE. **(D)** Re-evaluation of novel classification by PCA.

**FIGURE 4:**
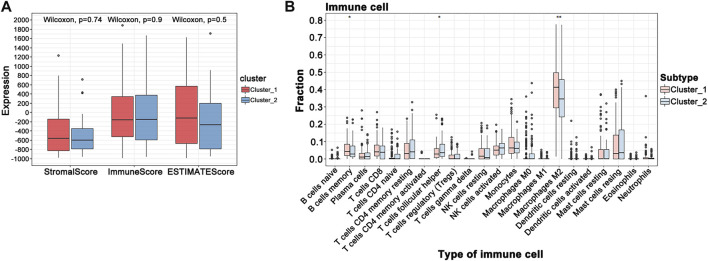
GBM tumor immune environment. **(A)** ESTIMATE algorithm analysis of clusters. **(B)** CIBERSORT analysis of clusters (***p < 0.001; **p < 0.01; *p < 0.05).

When the optimal k value was three, the consensus matrix showed a relatively sharp clear boundary, indicating stable and robust clustering (Figure 3A and Supplementary Figure S3). Owing to the relatively small sample size in cluster 3, we divided the patients into two clusters after excluding the samples in cluster 3. Interestingly all methods—NMDS1, tSNE, and PCA—verified subclass stability by consensus subtype clustering (Figures 3B–D). We used the ESTIMATE algorithm to explore the relationship between immune cell infiltration and the two subgroups and failed to find differences in immune scores across multiple classifications (Figure 4A). Furthermore, an assessment of the prevalence of twenty-two immune cell subtypes revealed that M2 macrophages were obviously elevated in cluster 1 relative to cluster 2 (Figure 4B).

### New classification establishment and evaluation based on WGCNA

**FIGURE 5:**
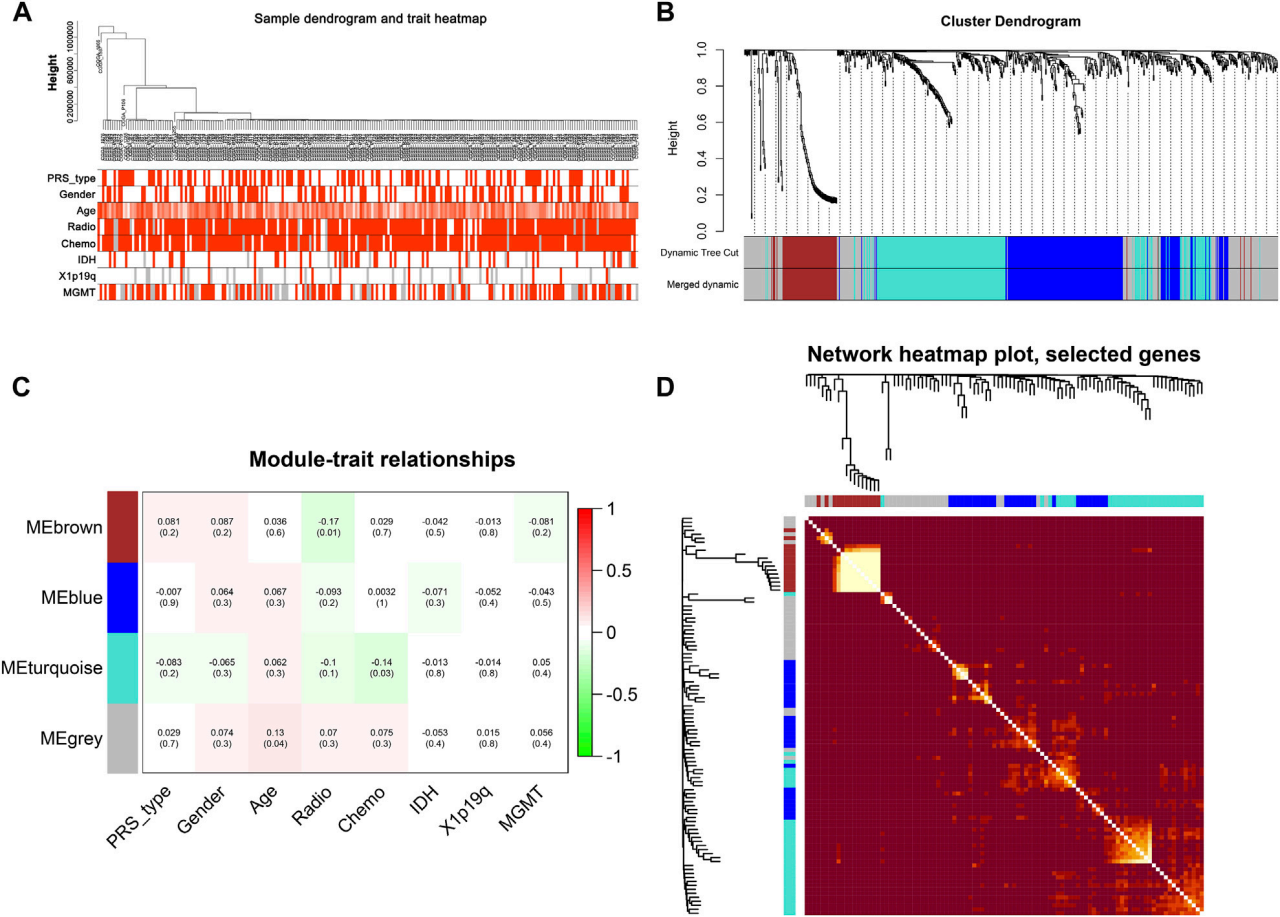
New subgroups based on the WGCNA analysis. **(A)** Sample GBM dendrogram and trait heat map. **(B)** GBM cluster dendrogram. **(C)** Module-trait relationships of WGCNA. **(D)** Display of network heat map plot and selected genes.

**FIGURE 6:**
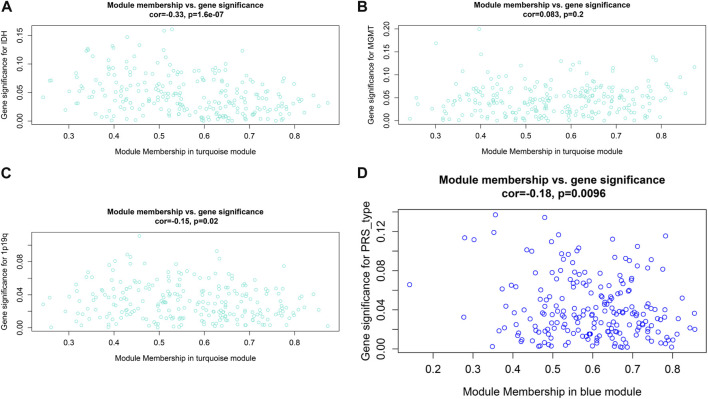
Relationship between modules and clinical features. **(A)** Relationship between IDH status and turquoise module. **(B)** Relationship between MGMT status and turquoise module. **(C)** Relationship between 1p19q status and turquoise module. **(D)** Relationship between PRS type and blue module.

After data preprocessing, we obtained the gene expression matrix, and a sample clustering tree was drawn to visualize distribution, using a soft threshold of 10 (R^2^ = 0.63) to construct a scale-free network (Figure 5A), which may exclude biased samples. Next, adjacency and topological overlap matrices were built for further analyses. Subsequently, a dendrogram of all differentially expressed gene clusters was established based on dissimilarity (Figure 5B). As shown in Figure 6C, the blue module was negatively related to PRS type, and the turquoise module was negatively correlated with *IDH* and 1p19q status (Figure 5C). Thus, these two modules were selected as clinically important modules. The co-relationships among these clinical modules are presented in Figure 6D. We also performed correlation analyses between important clinical features and the selected co-expression modules (Figures 6A–D). Based on the previous risk model, we identified eight genes (*ANKRD20A4, CLOCK, CNTRL, ICA1, LARP4B, RASA2, RPS6*, and *SET*) in the blue module and three genes (*MSH2, ZBTB34*, and *DDX31*) in the turquoise module.

### Hub prognostic gene selection and evaluation

**FIGURE 7:**
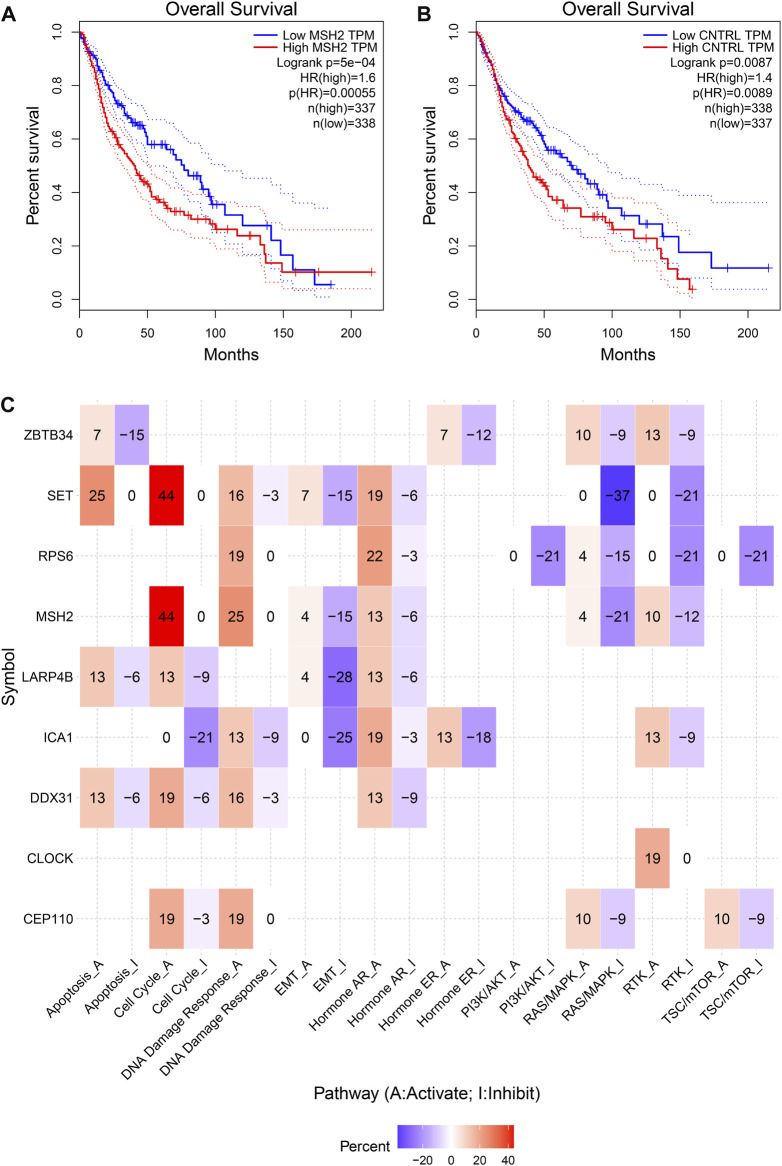
Hub prognostic genes selection. **(A)** Kaplan–Meier curve of MSH2 based on the TCGA dataset. **(B)** Kaplan–Meier curve of CNTRL based on the TCGA dataset. **(C)** MSH2 pathways based on GSCALite.

**FIGURE 8:**
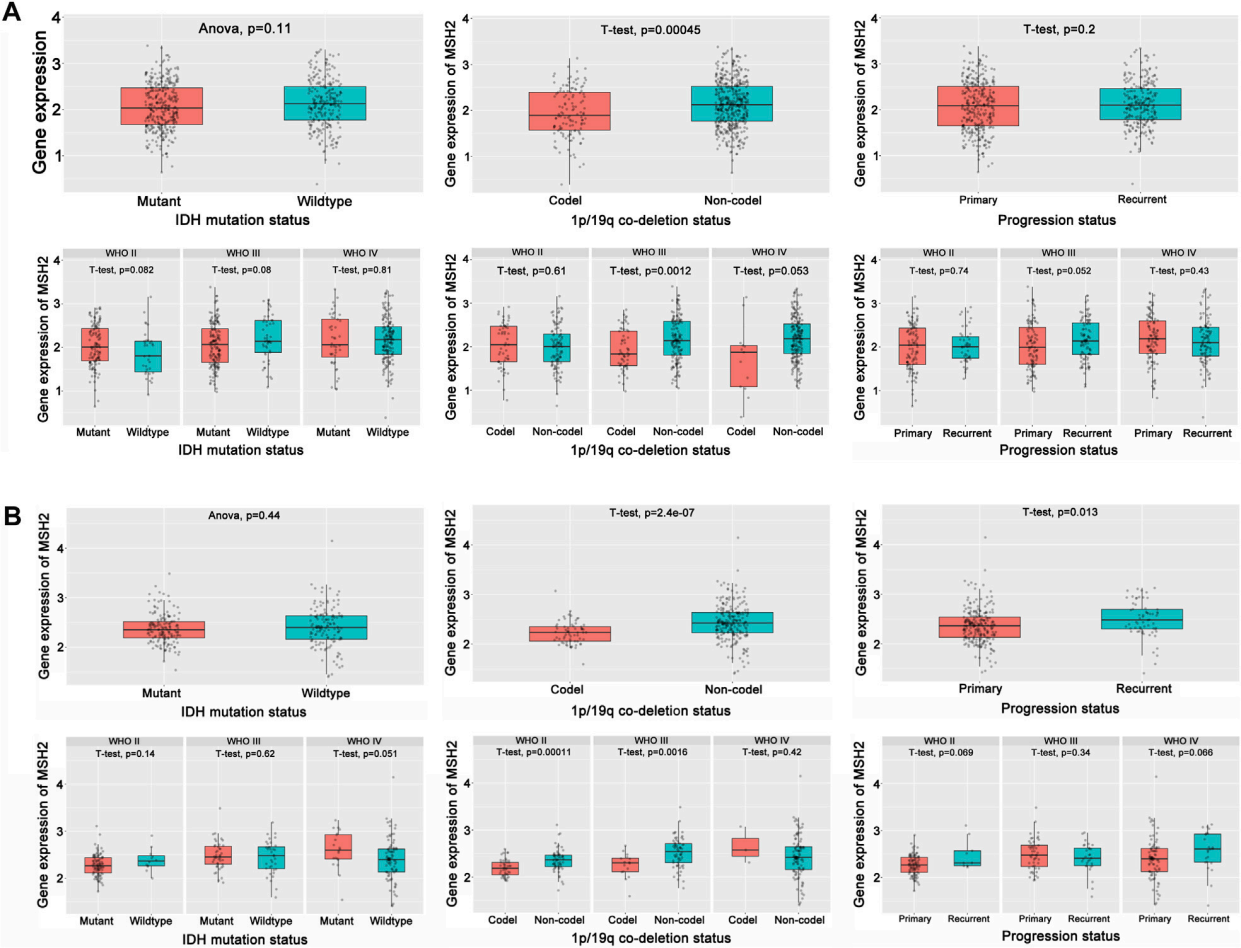
Hub prognostic genes evaluation. **(A)** Role of MSH2 in the CGGA693 cohort. **(B)** Role of MSH2 in the CGGA325 cohort.

**FIGURE 9:**
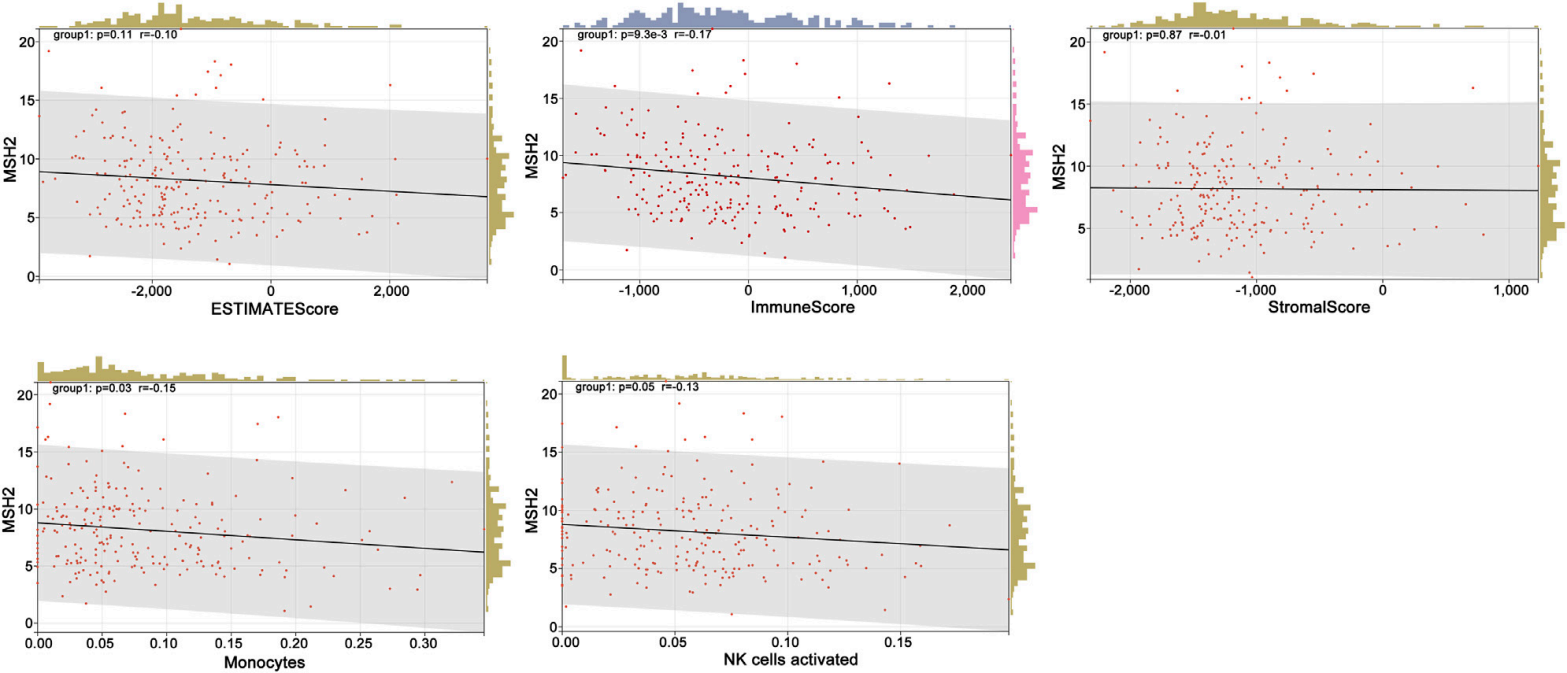
Relationship between MSH2, immune score, and cells.

To further explore the innate mechanism and determine hub genes in GBM, we utilized a publicly available website to test the survival rate of these 11 expression levels in patients with GBM. The KM curve suggested that increased *MSH2* and *CNTRL* were significantly associated with poorer OS, using median expression as a cut-off value (*p* < 0.05) (Figures 7A,B). Based on GSCALite, we observed that *MSH2* may activate the cell cycle and DNA damage response and inhibit epithelial–mesenchymal transition (EMT) and *RAS/MAPK* signaling. However, we failed to reveal the pathways involved in *CNTRL* (Figure 7C). Therefore, *MSH2* was comprehensively evaluated as the most important biomarker, using the CGGA dataset. In the CGGA693 RNA-seq cohort, we observed that *MSH2* was elevated relative to all glioma patients in patients lacking the 1p/19q co-deletion. MSH2 was particularly elevated in patients with WHO III or IV pathology (Figure 8A). In the CGGA325 cohort, *MSH2* was also higher in patients lacking the 1p/19q co-deletion, particularly in those diagnosed as WHO grade II or III. Specifically, *MSH2* may be positively associated with recurrence (Figure 8B). Considering the bright prospects of immunology, we showed that the expression of *MSH2* was negatively correlated with the immune score, and the levels of monocytes and NK cells were elevated (Figure 9).

### Effect of *MSH2* silencing on proliferation, migration, and invasion of GBM

**FIGURE 10:**
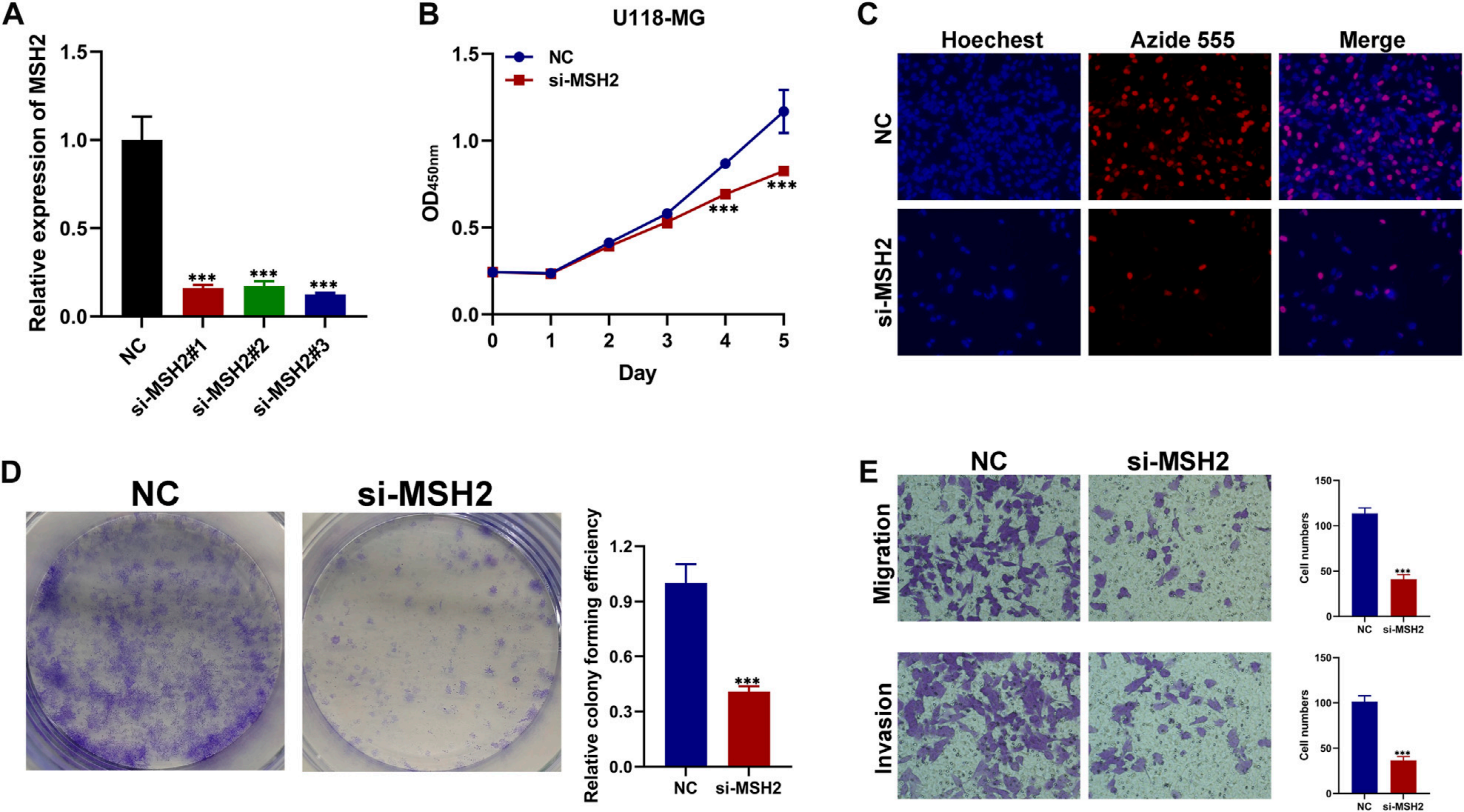
Biological functional validation of MSH2 in GBM cell. **(A)** Silencing efficiency of MSH2. CCK8 assay **(B)**, EDU assay **(C)**, and colony formation assay **(D)** showing the effect of MSH2 knockdown on proliferation of U-118 MG cells. **(E)** Transwell assay indicating that MSH2 depletion markedly weakens migration and invasion by U-118 MG cells (*p < 0.05, **p < 0.01, and ***p < 0.001, *n* = 3).

We further investigated the function of the hub genes in GBM development and progression. To further verify the reliability and accuracy of our diagnostic model, we selected *MSH2* after a careful literature review revealed that it has rarely been studied in GBM. Subsequently, siRNAs targeting *MSH2* were designed and synthesized. Compared to NC-transfected cells, *MSH2* expression was lower in cells transfected with si-MSH2 (Figure 10A), and since the silencing efficacy of si-MSH2#3 (termed si-MSH2) is the highest, we have chosen it for further study. CCK8 and EdU assays showed that *MSH2* depletion significantly suppressed GBM cell growth (Figures 10B,C). Similarly, the proliferative capacity of GBM cells was strikingly suppressed after silencing *MSH2* by clone formation (Figure 10D), indicating that *MSH2* plays an important role in the growth of GBM. The results of the Transwell assay revealed that MSH2 knockdown markedly attenuated GBM cell migration and invasion (Figure 10E).

## Discussion

Glioblastoma is an aggressive intrinsic brain tumor that may occur at any age and has a high incidence of recurrence. Surgery followed by involved-field radiotherapy in combination with temozolomide chemotherapy constitutes the standard therapy for newly diagnosed GBM patients (Stupp et al., 2005). Simultaneously, an emerging area of interest in cancer therapy, notably for GBM, has promoted the discovery of a variety of targeted therapies. For example, alkylating agent chemotherapy, and aberrant CpG methylation of the promoter region of the O6-methylguanine DNA methyltransferase (MGMT) gene has been confirmed to have a predictive value for patients using temozolomide (Hegi et al., 2005; Hegi et al., 2019). However, no targeted therapeutic intervention has been shown to prolong overall survival or to be superior to current treatments for GBM.

In this study, filtering analysis was performed from the CGGA and TCGA datasets (only GBM) and multiple key genes, including *MSH2* and *CNTRL* were identified. KM survival analysis, univariate Cox analysis, and multivariate Cox analysis validated the predictive value of these genes. To visualize the practicability of these genes, LASSO analysis was performed to calculate the risk score and construct a risk model. Interestingly, the risk score may be an independent biomarker to evaluate clinical efficacy, which is superior to other clinical features. Notably, multiple analyses have suggested that these potential molecules might function as regulators in cancer pathogenesis, but how they act in an mRNA-interactive manner during GBM progression remains unknown. Thus, we used unsupervised classification to classify patients into specific groups with potential gene co-expression. Moreover, we utilized WGCNA to identify the relationship between the co-expression of genes and clinical features. Taken together, our data support that *MSH2* plays a key role in GBM progression.

The *MSH2* gene, which plays a key role in DNA mismatch repair (*MMR*), may form a complex with BLM-p53-RAD51 in response to DNA damage repair (Yurgelun et al., 2017; Xu et al., 2021). When DNA is damaged, *MSH2* promotes apoptosis by regulating *ATR/Chk2/p53* signal transduction (Yang et al., 2004). *MSH2* is intimately linked to the occurrence and development of multiple cancers. In multiple gastric cancers (MGC) characterized by the presence of more than two different tumors in the stomach, the *MSH2* mutations, particularly germline *MSH2 X314*_splice variants, may contribute to carcinogenesis, suggesting the consideration of more radical surgery and/or anti-PD-1/PD-L1 therapy (Wang et al., 2020). In oral diseases, the study supports that patients with *MSH2* overexpression may easily present with oral squamous cell carcinoma (Donís et al., 2021). Moreover, *MSH2* mutation was found in 31% of patients with Lynch syndrome-associated GBMs, indicating that *MSH2* may play important role in the progression of GBM (Kim et al., 2022). In this study, to verify the accuracy of our prognostic model, the relationship between *MSH2* expression and clinical characteristics was evaluated using WGCNA. We found that *MSH2* plays the key role in the turquoise module, and is negatively associated with IDH and 1p19q status. Furthermore, gene set enrichment analysis (GSEA) revealed a role of *MSH2* in tumor progression, which is generally enriched in activation of the cell cycle, DNA damage response, and inhibition of EMT and *RAS/MAPK* pathways. We further investigated the function of the hub genes in GBM development and progression. To verify the reliability and accuracy of our diagnostic model, we performed several experiments and demonstrated that depletion of *MSH2* significantly suppressed proliferation, and weakened the migration and invasion ability of GBM cells, indicating that *MSH2* is a prognostic and therapeutic target for GBM.

## Conclusion

Our study comprehensively analyzed the CGGA datasets of GBM and constructed a 17-gene risk signature using LASSO regression analysis. Furthermore, we developed a novel classification using ConsensusClusterPlus based on these 17 genes and evaluated the tumor immune environment using ESTIMATE and CIBERSORT. Further, by applying WGCNA analysis, we identified eight and three genes from the blue and turquoise modules, respectively. Finally, through validation of public websites and experiments, *MSH2* was verified as the hub biomarker.

## Data Availability

The datasets for this article are not publicly available due to concerns regarding participant/patient anonymity. Requests to access the datasets should be directed to the corresponding author.
