# Supplementary material for: Comprehensive development and validation of gene signature for predicting survival in patients with glioblastoma
Source: Front Genet. 2022 Aug 10;13:900911. doi: 10.3389/fgene.2022.900911 (PMC9399759; doi:10.3389/fgene.2022.900911)
Supplement: Supplementary file 3 [file Table1.DOCX]

|  | **CGGA693** | **CGGA325** | ***p*-value** |
| --- | --- | --- | --- |
| **Toal** | 235 | 138 |  |
| **Age** | 48.94±13.81 | 46.61±12.56 | 0.1048 |
| **PRS Type** |  |  | 0.2777 |
| Primary | 132 | 85 |  |
| Recurrent | 103 | 52 |  |
| **Gender** |  |  | 0.4418 |
| Male | 139 | 87 |  |
| Female | 96 | 50 |  |
| **Radio status** |  |  | 0.0209* |
| Untreated | 31 | 32 |  |
| Treated | 192 | 100 |  |
| Unknown | 12 | 5 |  |
| **Chemo status** |  |  | 0.0011** |
| Untreated | 26 | 34 |  |
| Treated | 198 | 99 |  |
| Unknown | 11 | 4 |  |
| **IDH mutation** |  |  | 0.0542 |
| Wildtype | 181 | 98 |  |
| Mutant | 44 | 39 |  |
| Unknown | 10 | 0 |  |
| **1p19q codeletion** |  |  | >0.9999 |
| Non-codel | 195 | 127 |  |
| Codel | 12 | 7 |  |
| Unknown | 28 | 3 |  |
| **MGMTp_methylation** |  |  | 0.3112 |
| Un-methylated | 87 | 70 |  |
| Methylated | 104 | 65 |  |
| Unknown | 44 | 2 |  |
