# Supplementary material for: Comprehensive development and validation of gene signature for predicting survival in patients with glioblastoma
Source: Front Genet. 2022 Aug 10;13:900911. doi: 10.3389/fgene.2022.900911 (PMC9399759; doi:10.3389/fgene.2022.900911)
Supplement: Supplementary file 4 [file Table4.DOCX]

**Supplementary Table S4:** The sequences of primers and siRNA used in this study

| **The sequences of primers for PCR (5′→3′)** | |
| --- | --- |
| GAPDH | Forward: GTCAGCCGCATCTTCTTT |
| GAPDH | Reverse: CGCCCAATACGACCAAAT |
| MSH2 | Forward: TCACCTGAATGACATCTACCTCC |
| MSH2 | Reverse: GTCACCTGCCACTATTTCCTCC |
| **The sequences of siRNAs (5′→3′)** | |
| NC | Sense: UUCUCCGAACGUGUCACGU |
| NC | Antisense: ACGUGACACGUUCGGAGAA |
| si-MSH2#1 | Sense:CUUGCUGAAUAAGUGUAAATT |
| si-MSH2#1  si-MSH2#2  si-MSH2#2 | Antisense: UUUACACUUAUUCAGCAAGGC  Sense: UCAUGUUGCAGAGCUUGCUTT  Antisense: AGCAAGCUCUGCAACAUGAAU |
| si-MSH2#3 | Sense: GGAUUAAGCAGCCUCUCAUTT |
| si-MSH2#3 | Antisense: AUGAGAGGCUGCUUAAUCCTT |
